# Production and Characterization of Monoclonal Antibodies against Human Nuclear Protein FAM76B

**DOI:** 10.1371/journal.pone.0152237

**Published:** 2016-03-28

**Authors:** Xiaojing Zheng, Yanqing Li, Junli Zhao, Dongyang Wang, Haibin Xia, Qinwen Mao

**Affiliations:** 1 Co-Innovation Center for Qinba Regions’ Sustainable Development, College of Life Sciences, Shaanxi Normal University, Xi’an, 710062, Shaanxi, P. R. China; 2 Department of Pathology, Northwestern University Feinberg School of Medicine, Chicago, Illinois, 60611, United States of America; The Scripps Research Institute, UNITED STATES

## Abstract

Human FAM76B (hFAM76B) is a 39 kDa protein that contains homopolymeric histidine tracts, a targeting signal for nuclear speckles. FAM76B is highly conserved among different species, suggesting that it may play an important physiological role in normal cellular functions. However, a lack of appropriate tools has hampered study of this potentially important protein. To facilitate research into the biological function(s) of FAM76B, murine monoclonal antibodies (MAbs) against hFAM76B were generated by using purified, prokaryotically expressed hFAM76B protein. Six strains of MAbs specific for hFAM76B were obtained and characterized. The specificity of MAbs was validated by using FAM76B^-/-^ HEK 293 cell line. Double immunofluorescence followed by laser confocal microscopy confirmed the nuclear speckle localization of hFAM76B, and the specific domains recognized by different MAbs were further elucidated by Western blot. Due to the high conservation of protein sequences between mouse and human FAM76B, MAbs against hFAM76B were shown to react with mouse FAM76B (mFAM76B) specifically. Lastly, FAM76B was found to be expressed in the normal tissues of most human organs, though to different extents. The MAbs produced in this study should provide a useful tool for investigating the biological function(s) of FAM76B.

## Introduction

Human FAM76B (hFAM76B) is a 39 kDa nuclear speckle-localized protein that consists of 339 amino acids (NP_653265; hypothetical protein LOC143684). It contains homopolymeric histidine tracts that are considered a targeting signal for nuclear speckles [1,2,3]. Although the function of FAM76B is still unknown, many poly(His)-containing proteins have been shown to endow DNA- and RNA-related functions and are overrepresented in the nervous systems’ development [3].

In order to facilitate the functional study of FAM76B, we generated anti-hFAM76B monoclonal antibodies (MAbs) by using hFAM76B-6His fusion protein expressed in *Escherichia coli* BL21. Six strains of MAbs specific for hFAM76B were obtained and further characterized by using enzyme-linked immunosorbent assays (ELISAs), Western blot, immunoprecipitation (IP) and immunohistochemical staining (IHC). These anti-hFAM76B MAbs should help researchers explore the biological function(s) of FAM76B in future studies.

## Materials and Methods

### Cell culture

HepG2 (human hepatocellular liver carcinoma cell line), Shsy5y (human neuroblastoma cell line), HEK293 (human embryonic kidney cell line), NIH/3T3 (mouse embryo fibroblast cell line), Hepa1-6 (mouse hepatocellular liver carcinoma cell) and SP2/0 (mouse myeloma cell line) were obtained from American Type Culture Collection (ATCC, Manassas, VA). All cells were cultured in Dulbecco’s Modified Eagle’s Medium (Gibco, Grand Island, NY) supplemented with 10% (vol/vol) fetal bovine serum (Gibco, Grand Island, NY), 1% Penicillin/Streptomycin (P/S) and 1% L-glutamate, and maintained in a humidified chamber with 5% CO_2_ at 37°C.

### Generation of FAM76B^-/-^ HEK 293 cell line

To build FAM76B^-/-^ HEK 293 cell line, the primers for four single guide RNAs (sgRNAs) targeting the Exon 1 and Exon4 of the human FAM76B gene were designed, then synthesized by BGI (Beijing Genomics Institute, Beijing, China). The corresponding sequences of sgRNA were shown in supporting information (S1 Table). The four sgRNA oligonucleotides were annealed, and then cloned into a pU6-sgRNA expressing vector. The resultant plasmids were named pU6-hFAM76B-sgRNA1, pU6-hFAM76B-sgRNA2, pU6-hFAM76B-sgRNA3 and pU6-hFAM76B-sgRNA4, respectively. The sgRNA4 was shown to have best activity by T7 endonuclease I (7TEI) assay. Then pU6-hFAM76B-sgRNA4 was co-transfected into HEK 293 cells with pCMV-Cas9. Through several rounds of dilution cloning and PCR diagnosis, the FAM76B^-/-^ HEK 293 cell line was obtained. The sequence results demonstrated that two alleles of FAM76B from FAM76B^-/-^ HEK 293 cell line were mutated by the insertion of 250 bp and 118 bp into the cutting site of the genome respectively.

### Plasmid construction

The human full-length FAM76B cDNA was amplified based on the template of the MegaMan Human Transcriptome Library (Agilent-Stratagene, Santa Clara, CA) by nested PCR using the following primers, the first pair of primers, forward 5’-AGGGGGAGGGGGAGGAGGAG-3’, and reverse 5’-AAAAACCCTGCTGCTCTGAC-3’, the second pair of primers (nested primers), forward 5’-AATCGATATGGCGGCCT CGGCCCTG-3’ and reverse 5’-ATCTAGATTAAGGAGATGTTAGTAT-3’. The amplified products were gel-purified and cloned into the pGEM-T easy Vector (Promega, Madison, WI). The positive clone confirmed by restriction enzyme digestion and sequencing was named pGEMT-hFAM76B. Then the human full-length FAM76B was cloned into the *Cla* I/*Spe* I sites of the pRSET-B vector (Invitrogen, Carlsbad, CA); the obtained plasmid was called pRSET-hFAM76B. The human full-length FAM76B without stop codon was amplified and cloned into pAd5 E1-CMV-MCS-TAA and pAd5 E1-CMV-MCS-Flag. The resultant plasmids were named pAd5-E1-CMV-hFAM76B-TAA and pAd5-E1-CMV-hFAM76B-Flag, respectively. Using a similar strategy, the coding region of mouse full-length FAM76B without stop codon was amplified by PCR based on the template of the pOTB7-mFAM76B vector (ATCC, Manassas, VA) and cloned into pAd5 E1-CMV-MCS-TAA and pAd5 E1-CMV-MCS-Flag. The resultant plasmids were named pAd5-E1-CMV-mFAM76B–TAA and pAd5-E1-CMV-mFAM76B-Flag, respectively.

### Expression of the full-length and truncated FAM76B in *E*. *coli* BL21

Truncated hFAM76B mutants of different lengths were generated by PCR. The primers used for amplifying these fragments are provided in S1 Table. The amplified products were gel-purified and cloned into a pGEM-T easy Vector (Promega, Madison, WI). The positive clones were named pGEMT-FAM76B-FX (where X stands for 1, 2, 3, 4, 5 or 6). The six truncated hFAM76B fragments were cloned into a pRSET-B vector, respectively. The positive clones were named recombinant pRSET-hFAM76B-FX (where X stands for 1, 2, 3, 4, 5 or 6).

The pRSET-hFAM76B vector was transformed into *E*. *coli* BL21 (DE3), then a single colony of bacteria was inoculated into 10ml LB medium containing 30μg/mL kanamycin, which was grown overnight at 37°C with shaking. Then 4ml of overnight bacteria were inoculated into 400 mL of LB medium containing 30μg/mL kanamycin. When the cultures reached an optical density (O.D.) of 0.8 at 600 nm, the bacteria were induced by 1 mM IPTG for 4 to 5 hours. The bacterial pellets were then harvested, suspended in native lysis buffer, sonicated and centrifuged. The soluble fraction was subsequently used for purifying the recombinant hFAM76B protein with nitrilotriacetic acid (Ni–NTA) column (Qiagen, Valencia, CA). The purified hFAM76B proteins were stored at -80°C for subsequent experiments. The six truncated hFAM76B mutants were obtained using a similar strategy.

### Hybridoma generation

All animal protocols used in the study were approved by the Institutional Animal Care and Use Committee (IACUC) of Shaanxi Normal University. Four-week-old female Balb/c mice (n = 5) (Animal Center of The Fourth Military Medical University, Xi’an, China) were immunized with approximately 40 μg of purified hFAM76B-6His per mouse, every three weeks for a total of 3 doses. The immunization procedures were performed as follows. hFAM76B-6His protein in Freund’s complete adjuvant (Sigma–Aldrich, St. Louis, MO) was injected for the first immunization. Then hFAM76B-6His protein in Freund’s incomplete adjuvant (Sigma–Aldrich) was used as immunogen for the second and third immunization. Sera taken on day 0 were used as pre-immunized antibody. Seven days after the third immunization, blood samples were taken to determine antibody titer by indirect ELISA. Pre-immunized sera were used as negative control and 100μl of purified hFAM76B-6His (10μg/mL) were used for screening by ELISA. The mouse with high serum titers (more than 1:60,000), as determined by an ELISA test, was selected for fusion of spleen cells with myeloma cells. The mouse was boosted a fourth time before cell fusion. The fusion of SP2/0 myeloma cells with spleen cells isolated from immunized Balb/c mouse was carried out using standard methodology. The positive hybridoma cells were cloned at least three times by limiting dilution in aminopterin-free selection medium. The final positive hybridoma cells secreting antibodies against hFAM76B were stored in liquid nitrogen.

### ELISA and subclass of MAbs

ELISA was performed on 96-well plates, which were coated with hFAM76B-6His fusion protein overnight at 4°C and then blocked by incubation with 10 mg/ml BSA. MAbs were applied to duplicate wells, followed by HRP-conjugated goat anti-mouse IgG (Thermo Fisher Scientific, Waltham, MA) as the secondary antibody. ABTS [2, 2'-Azino-bis (3-ethylbenzothiazoline-6-sulfonic acid ammonium salt)] (Thermo Fisher Scientific, Waltham, MA) was added subsequently and the absorbance was recorded at 490 nm using a microplate reader (Thermo Fisher Scientific, Waltham, MA). An immunoglobulin class/subclass test was performed using a mouse monoclonal antibody isotyping kit (Sigma-Aldrich, St. Louis, MO).

### Western blot

HEK 293 cells were transiently transfected with an expressing vector carrying hFAM76B-Flag, hFAM76B, mFAM76B-Flag or mFAM76B by the standard calcium phosphate method. All transfected cells were grown at 37°C with 5% CO2. Forty-eight hours post-transfection, the cell pellets were collected and lysed in RIPA Lysis Buffer [50 mM Tris pH 7.5, 150 mM NaCl, 1% Nonidet P-40, 0.1% SDS, 1 mM phenylmethanesulfonyl fluoride (PMSF)] for 40 minutes on ice. Then the supernatants were collected and used for the whole-cell extracts by centrifugation. The generated whole-cell extracts were used for the source of recombinant protein. The whole-cell extracts from HepG2 cells, Shsy5y cells, NIH/3T3 cells, Hepa1-6 cells, HEK293 cells or FAM76B^-/-^ HEK 293 cells were obtained by similar methods for the resource of endogenous FAM76B protein. Recombinant proteins or endogenous FAM76B from the whole-cell extracts, and prokaryotically expressed proteins from an expressing vector carrying different length of hFAM76B were applied to SDS-PAGE, and subsequently blotted onto methanol pretreated polyvinylidene difluoride (PVDF) membranes (Millipore, Temecula, CA). The PVDF membranes were incubated with anti-FAM76B MAbs overnight at 4°C. Membranes were washed and incubated with HRP-conjugated goat anti-mouse IgG antibody. The membranes were then washed with Phosphate Buffered Saline with Tween 20 (PBST) and visualized using enhanced chemiluminescence (ECL) Western blot detection reagents (Thermo Scientific, Waltham, MA), according to the manufacturer’s protocol. Rabbit polyclonal anti-hFAM76B antibody (Sigma-Aldrich, St. Louis, MO) was used as a positive control in the experiments.

### Immunoprecipitation

MAbs were pre-bound to blocked protein G agarose. After washing, the antibody-resin complexes were incubated with whole-cell extracts of HEK 293 cells transfected with hFAM76B-Flag plasmids, which were prepared using similar methods as described in the section of western blot, and then eluted Immunoprecipitates dissolved in 2×SDS loading buffer were analyzed by SDS-polyacrylamide gel electrophoresis and immunoblotting.

### Immunofluorescence and immunohistochemistry

For the immunofluorescent detection of overexpressed hFAM76B and mFAM76B, HEK 293 cells were transiently transfected with pAd5-E1-CMV-hFAM76B-Flag or pAd5-E1-CMV-mFAM76B-Flag expression plasmids, as described above. Twenty-four hours post-transfection, cells were fixed with 4% paraformaldehyde in PBS for 10 min at room temperature, then cells were permeabilized with 1% BSA and 0.5%Triton X-100 in PBS and after washing, were incubated with purified anti-FAM76B MAbs (1:100) overnight at 4°C. The cells were then incubated with TRITC-conjugated goat anti-mouse IgG (1:100, Zhong Shan, Beijing, China), followed by image capture using a Zeiss Axio Observer Z1 fluorescence microscope. For the detection of endogenous hFAM76B and mFAM76B, HepG2, Shsy5y, FAM76B^-/-^ HEK 293 cells, NIH/3T3 and Hepa1-6 cells were fixed, washed and incubated with 3% H_2_O_2_ for 15 min at room temperature. Then the cells were incubated with purified anti-hFAM76B MAbs, followed by biotinylated-conjugated horse anti-mouse IgG (1:200, Vector Laboratories, Burlingame, CA). Lastly, the cells were incubated with 1% Avidin- biotinylated HRP complex, and visualized with DAB as the chromagen. The images were captured using a Leica DM IL LED microscope. Experiments were done in triplicate and were repeated at least twice.

To evaluate the FAM76B expression in human tissues, postmortem brain tissue, and peripheral tissues from the liver, spleen, kidney, heart, lung and adrenal gland were collected from four normal donors. The collection of postmortem samples has been approved by the Ethics Committee of the University. The consents for research were signed by the patients themselves or by their next of kin in their name. The tissues were fixed in 10% buffered formalin, paraffin embedded and cut in 5 mm sections followed by immunohistochemical stains with anti-hFAM76B MAb No.2 in a protocol similar to that described above.

### Confocal microscopy study

HEK 293 cells were transiently transfected with pAd5-E1-CMV-hFAM76B-Flag. Forty-eight hours post-transfection, the cells were fixed with 4% paraformaldehyde. After washing, the fixed cells were incubated with rabbit anti-SC35 antibody (1:1000; Abcam, Cambridge, MA) and MAb against hFAM76B No.2 (1:100) overnight at 4°C. After extensive washing, cells were incubated with TRITC-conjugated goat anti-mouse IgG (1:100, Zhong Shan, Beijing, China) and FITC-conjugated goat anti-rabbit IgG (1:100, Zhong Shan, Beijing, China). Lastly, cells were mounted and analyzed by confocal microscopy on an LSM 5 PASCAL laser scanning microscope.

## Results

### Expression and purification of recombinant hFAM76B

**Fig 1:**
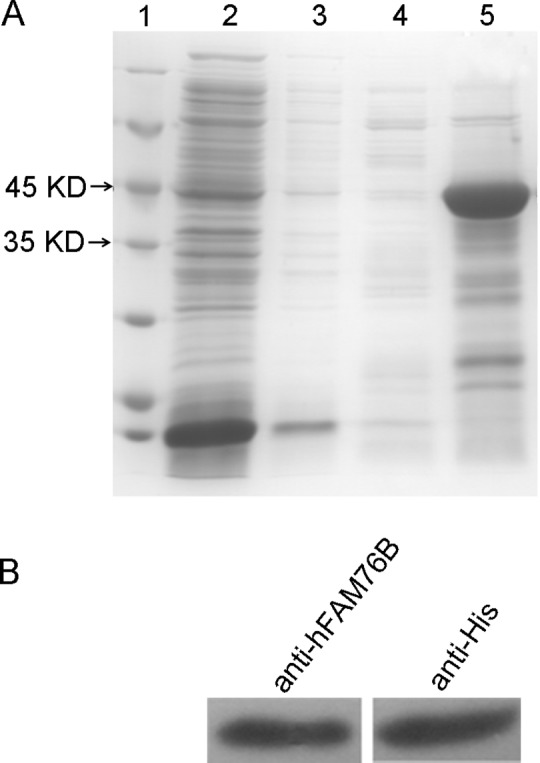
Production and purification of recombinant hFAM76B-6His. (A) His6-tagged hFAM76B protein was produced in *E*. *coli* BL21 (DE3), purified by Ni–NTA column and subjected to 10% SDS-PAGE. Coomassie blue staining revealed a band of 42 kDa, consistent with the predicted size of hFAM76B. Lane 1, protein molecular weight marker; lane 2, nonbound sample; lanes 3–5, samples eluted with 5 mM, 50 mM and 250 mM imidazole, respectively. (B) The purified protein (50 ng/lane) was confirmed by Western blot analysis with anti-6His antibody and rabbit polyclonal anti-hFAM76B antibody (Sigma, St. Louis, MO).

hFAM76B-6His fusion protein was expressed in *E*. *coli* BL21 (DE3) and then purified by Ni-NTA column. The eluted recombinant hFAM76B-6His proteins were subjected to 10% SDS-PAGE and visualized by coomassie blue staining, which revealed a major band with a molecular weight of approximately 42 kDa, consistent with the predicted size of hFAM76B (Fig 1A). The recombinant protein was further verified by Western blot with anti-His MAb and anti-hFAM76B rabbit polyclonal antibody (Fig 1B).

### Generation and validation of MAbs against hFAM76B

**Fig 2:**
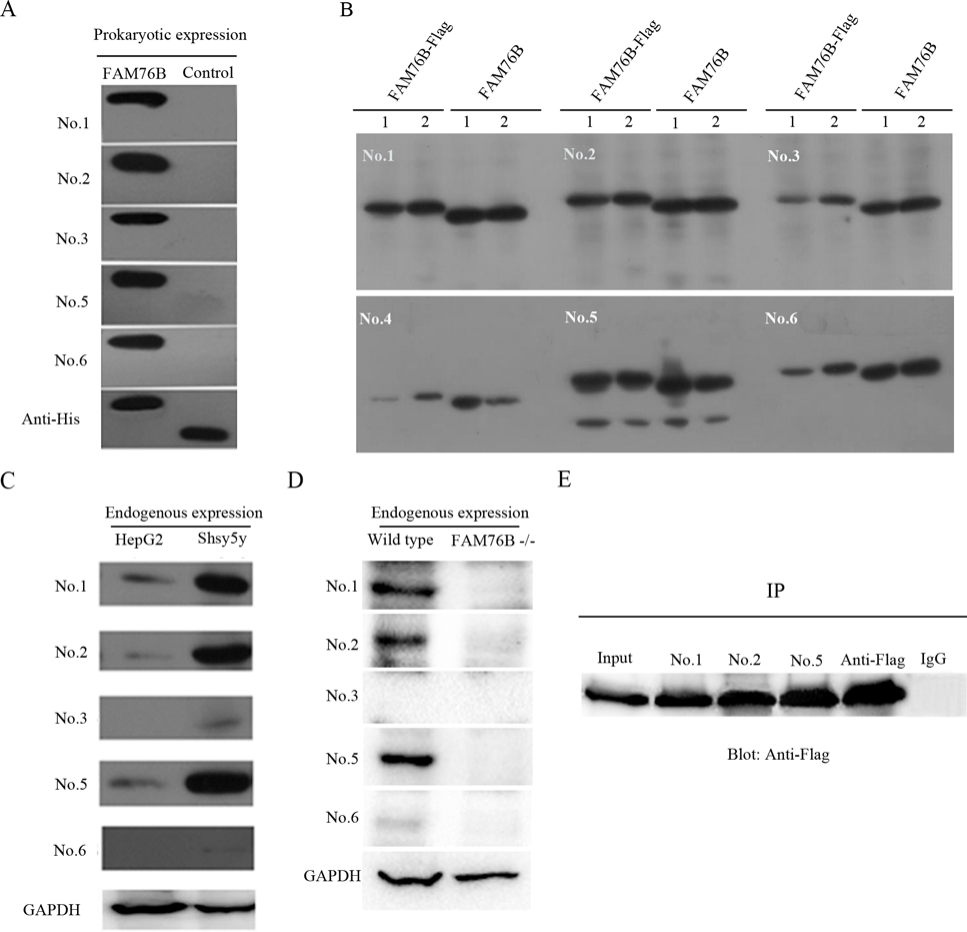
Western blotting and immunoprecipitation with MAbs against hFAM76B. (A) All MAbs except for No. 4 recognized hFAM76B-His recombinant protein expressed in *E*. *coli*. Truncated CD133-His fusion protein was used as the negative control protein and anti-His MAb was used as the positive control antibody. (B) Recombinant full-length hFAM76B from HEK 293 cells transfected with eukaryotic expressing vector carrying hFAM76B cDNA recognized by anti-FAM76B MAbs. 1and 2 indicated the samples from two independent transfection. (C) Endogenous FAM76B expressed in HepG2 or Shsy5y cells was detected by MAbs against hFAM76B; anti-GAPDH MAb was used as the positive control antibody. (D) Loss of FAM76B expression in FAM76B^-/-^ HEK 293 cells revealed by MAbs against hFAM76B; anti-GAPDH MAb was used for loading control. (E) The cell lysates of HEK 293 cells overexpressing hFAM76B-Flag were subjected to immunoprecipitation with anti-FAM76B MAbs followed by immunoblotting with anti-Flag MAb. Anti-Flag antibody was used as a positive control and rabbit anti-mouse IgG was used as a negative control.

Purified hFAM76B-6His was used to immunize mice for MAb production. The cell culture supernatants from the resulting hybridoma clones were screened by ELISA. At least six positive stable clones secreting anti-hFAM76B antibodies were obtained, all of which were IgG1. On the Western blot, all antibodies, except for No. 4, were able to sensitively detect prokaryotically expressed hFAM76B (Fig 2A), and overexpressed eukaryotic hFAM76B (Fig 2B) as well. An additional lower molecular weight band was detected by Mab No. 5 (Fig 2B), possibly corresponding to a degradation product of FAM76B protein. Three of the MAbs (No. 1, No. 2 and No. 5) had higher sensitivity to detect endogenous hFAM76B in HepG2 cells and Shsy5y cells than the other MAbs (Fig 2C). The specificity of MAbs was confirmed by using FAM76B^-/-^ HEK293 cells (Fig 2D).

In addition, these MAbs were tested by immunoprecipitation. hFAM76B-Flag fusion protein was overexpressed in HEK 293 cells, and whole-cell extracts were subjected to immunoprecipitation with different clones of anti-hFAM76B MAbs followed by immunoblotting with anti-Flag MAb. The results showed that anti-hFAM76B MAb No. 1, No. 2 and No. 5 were highly effective for immunoprecipitation (Fig 2E).

### Detection of hFAM76B expression by immunofluorescence and immunocytochemistry

**Fig 3:**
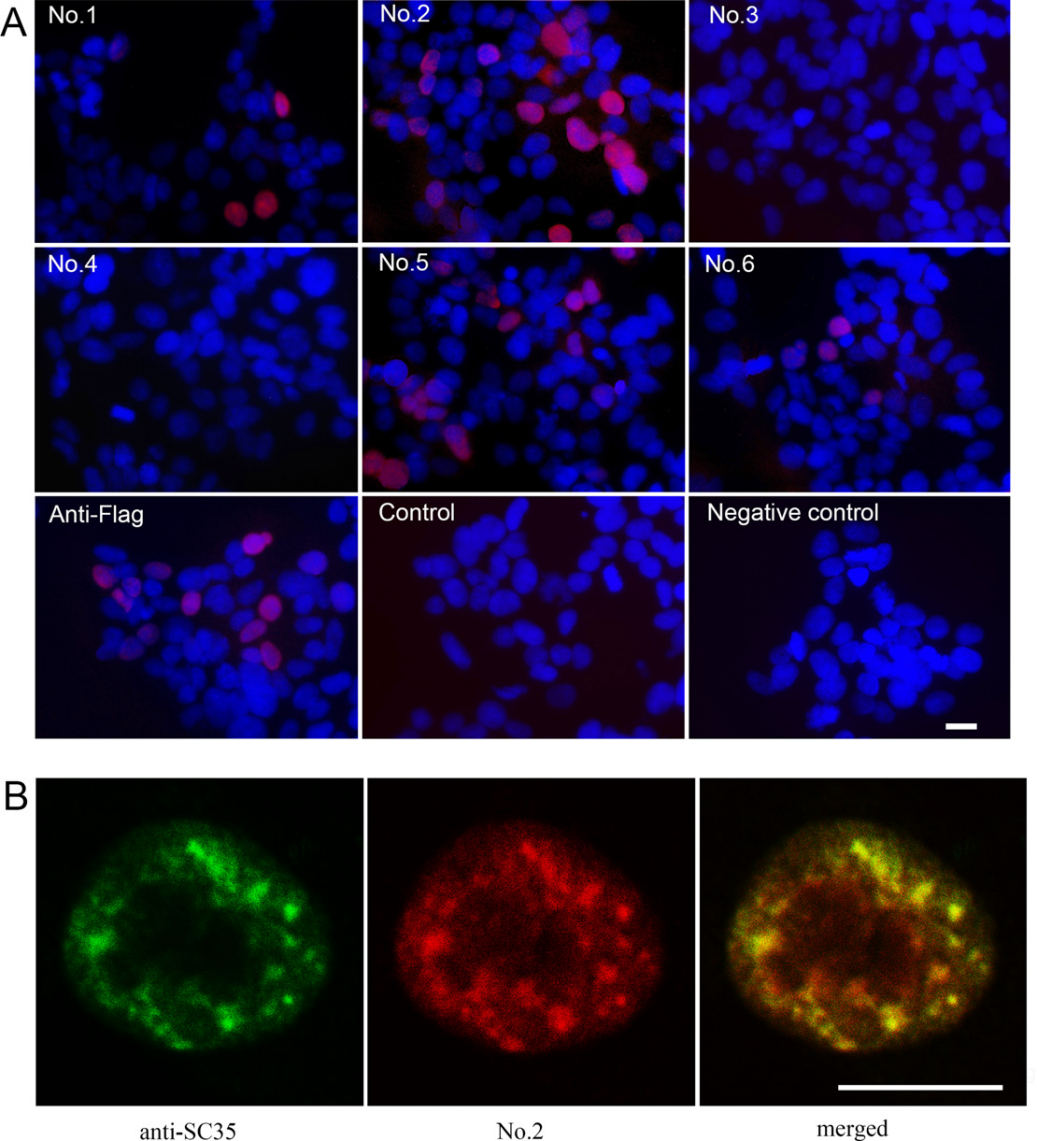
Intranuclear distribution of exogenous hFAM76B protein. (A) Immunofluorescent staining with anti-hFAM76B MAbs on HEK 293 cells overexpressing hFAM76B. HEK 293 cells were transfected with hFAM76B-Flag-expressing plasmid. Twenty-four hours after transfection, the cells were fixed with 4% paraformaldehyde and then stained with the anti-hFAM76B MAbs and an anti-Flag MAb. Normal mouse serum was used as a negative control. FAM76B^-/-^ HEK 293 cells were used for the negative control. TRITC-conjugated goat anti-mouse IgG was used as the secondary antibody. Nuclei were labeled with DAPI (blue). (B) Confocal study of hFAM76B and SC35, an endogenous marker of nuclear speckle compartments. HEK 293 cells were transfected with vector expressing hFAM76B and immunostained with anti-hFAM76B MAb No. 2 (red) and rabbit anti-SC-35 antibody (green). Scale bar = 10 μm.

HEK 293 cells were transiently transfected with hFAM76B-Flag expression vector and analyzed by indirect immunofluorescence for distribution of FAM76B. Four of the MAbs against hFAM76B (No. 1, No. 2, No. 5 and No. 6) revealed the nuclear localization of this protein with high intensity signal and low background noise (Fig 3A). FAM76B is a poly (His)-containing protein that accumulates in nuclear speckles. Confocal microscopy was used to detect colocalization of FAM76B with SC35, the endogenous marker of the nuclear speckle compartment [4]. The merge panel shows overlapping localization between FAM76B and SC35 (Fig 3B).

**Fig 4:**
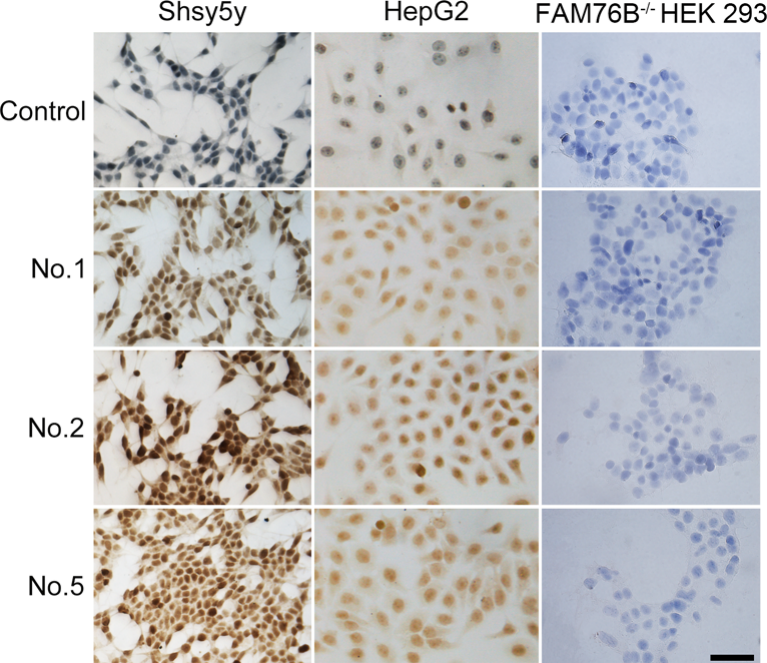
Immunocytochemical detection of endogenous hFAM76B. HepG2 and Shsy5y FAM76B^-/-^ HEK 293 cells were fixed with 4% paraformaldehyde and then stained with anti-FAM76B MAbs. Normal mouse serum was used as a negative control. Primary Abs were detected by a Vectastain ABC Kit with DAB as a substrate. Nuclei were labeled with hematoxylin. Scale bar = 50μm.

The endogenous expression of hFAM76B was evaluated by using human HepG2 and Shsy5y cell lines. FAM76B^-/-^ HEK 293 cells were used for negative control. The cells were fixed with 4% paraformaldehyde and then stained with different anti-hFAM76B MAbs. Antibody No. 1, No. 2 and No. 5 demonstrated strong endogenous expression of FAM76B in human HepG2 and Shsy5y cell lines with a nuclear distribution pattern (Fig 4).

### Dissection of the domains recognized by anti-hFAM76B MAbs

**Fig 5:**
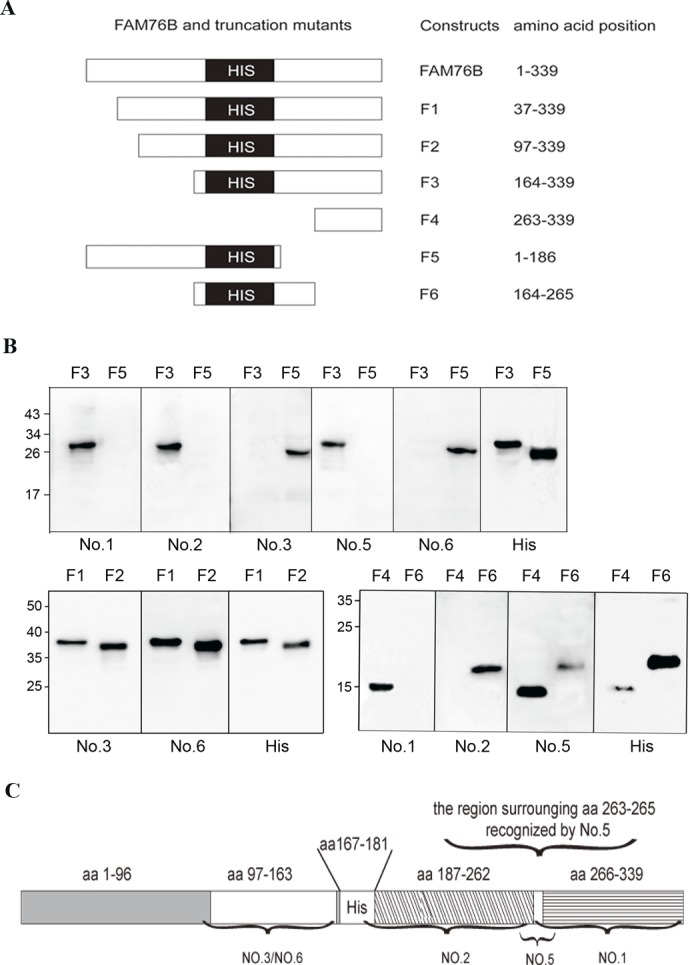
Domain mapping for anti-hFAM76B MAbs. (A) Cartoon of the position of the six different fragments in the FAM76B gene. (B) Western blot analysis of the binding of different anti-hFAM76B MAbs to full-length and six truncated mutants of hFAM76B under denaturing conditions. (C) Map of the domains recognized by different anti-hFAM76B MABs.

To identify the domains of different anti-hFAM76B MAbs, six truncated hFAM76B mutants (F1-F6) were expressed by plasmids. The position of the six different fragments in the FAM76B gene is illustrated in Fig 5A. These expressed proteins were separated by SDS–PAGE and analyzed by Western blot with different anti-hFAM76B MAbs (Fig 5B). Screening with FAM76B mutants F3 and F5 showed that anti-FAM76B MAb No. 3 and No. 6 recognized domains located on the N-terminal side of the protein before the polyhistidine repeat domain, while anti-FAM76B No. 1, 2 and 5 combined sequences after the polyhistidine repeat domain. Further, by using hFAM76B F1 and F2, MAb No. 3 and No. 6 were shown to be able to react with epitopes within aa97-163. Using hFAM76B F4 and F6, it was shown that MAb No. 2 and 1 recognized domains in aa187-262 and aa266-339, respectively. And the epitope recognized MAb No.5 is possibly located in the region surrounding aa263-265. The domains recognized by different anti-hFAM76B MAbs are shown in Fig 5C.

### MAbs against hFAM76B reacted with mouse FAM76B (mFAM76B)

**Fig 6:**
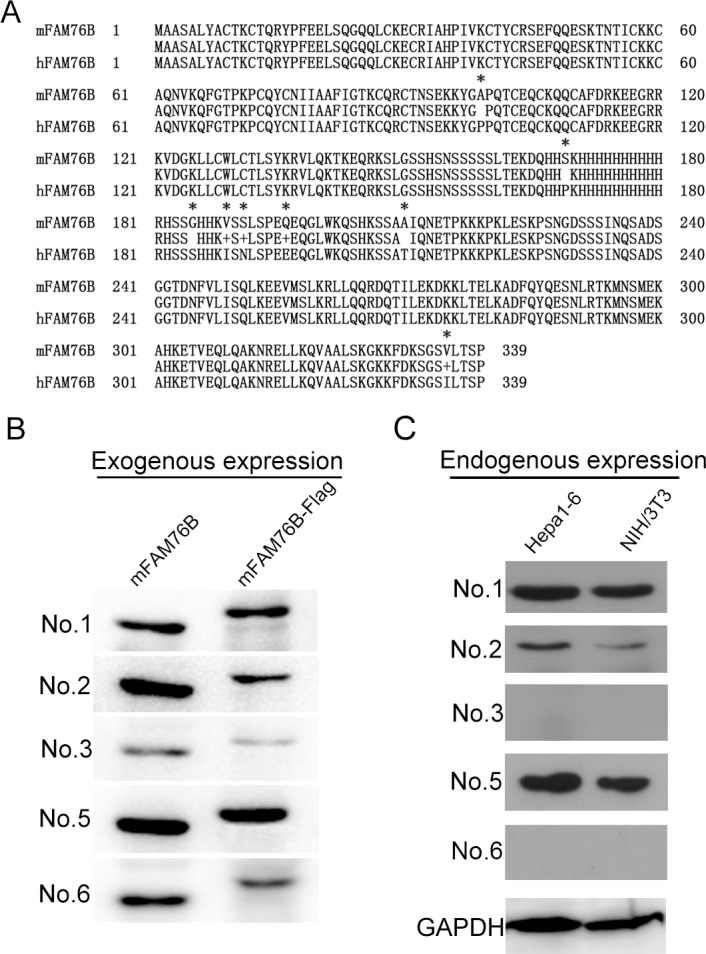
Mouse FAM76B recognized by MAbs against hFAM76B. (A) FAM76B human and mouse protein sequences were aligned using the Blast 2 Sequences program. Asterisks indicate different amino acids between these two sequences. (B) Exogenous overexpression of mFAM76B in HEK 293 cells was detected by Western blot with MAbs against hFAM76B. All MAbs except No. 4 reacted with the overexpressed mFAM76B sensitively and specifically. (C) Endogenous mFAM76B in NIH/3T3 and Hepa1-6 cells were revealed by Western blot using MAb No.1, 2 and 5.

**Fig 7:**
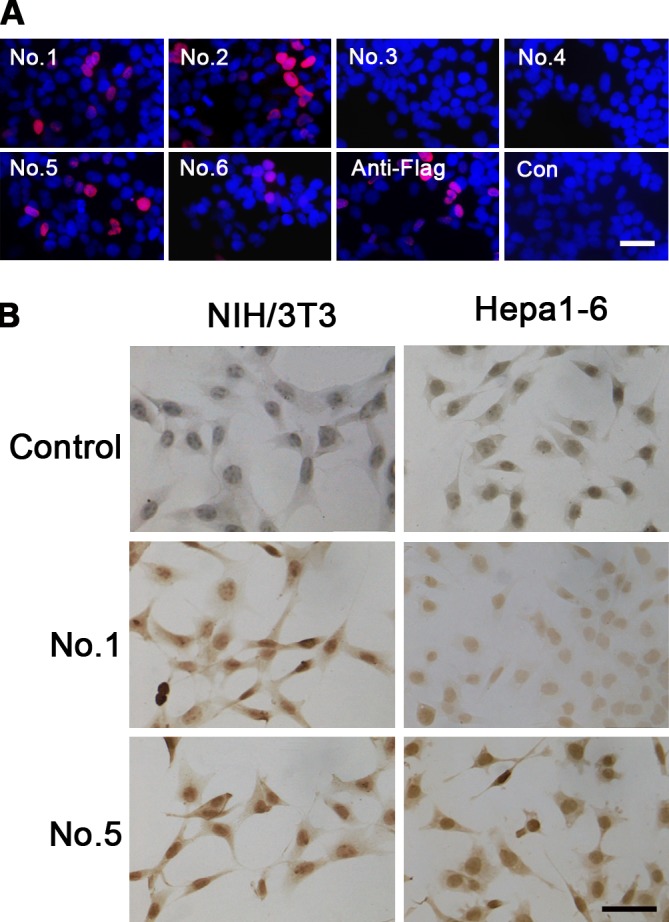
Exogenous and endogenous mFAM76B recognized by anti-hFAM76B MAbs. (A) Intranuclear localization of overexpressed mFAM76B in HEK 293 cells revealed by immunofluorescence staining. HEK 293 cells were transfected with plasmid expressing mFAM76B-Flag and stained with anti-hFAM76B MAbs and anti-Flag MAb. Normal mouse serum was used as a negative control. TRITC-conjugated goat anti-mouse IgG was used as the secondary antibody. Nuclei were labeled with DAPI (blue). (B) Endogenous mFAM76B revealed by immunohistochemical staining with MAbs against hFAM76B. NIH/3T3 and Hepa1-6 cells were fixed with 4% paraformaldehyde and then stained with the anti-hFAM76B MAbs. Normal mouse serum was used as a negative control. Primary Abs were detected by a Vectastain ABC Kit with DAB as a substrate. Nuclei were labeled with hematoxylin. Bar = 50 μm.

Protein sequence alignment and analysis of human and mouse FAM76B indicated a high degree of sequence conservation (Fig 6A). Hence, we reasoned that MAbs against hFAM76B would recognize mFAM76B specifically. Exogenous and endogenous mFAM76B were analyzed by Western blot using all six MAbs against hFAM76B. Similar to hFAM76B, all MAbs except No. 4 reacted with the overexpressed mFAM76B, sensitively and specifically. In addition, MAbs No. 1, 2 and 5 were more sensitive in recognizing endogenous mFAM76B than the other MAbs (Fig 6B and 6C). Immnofluorescent and immunohistochemical staining with different MAbs against hFAM76B demonstrated a strong and specific intranuclear distribution of overexpressed mFAM76B in HEK 293 cells, and endogenous mFAM76B in NIH/3T3 and Hepa1-6 cells (Fig 7).

### The expression of FAM76B in normal human organs

**Fig 8:**
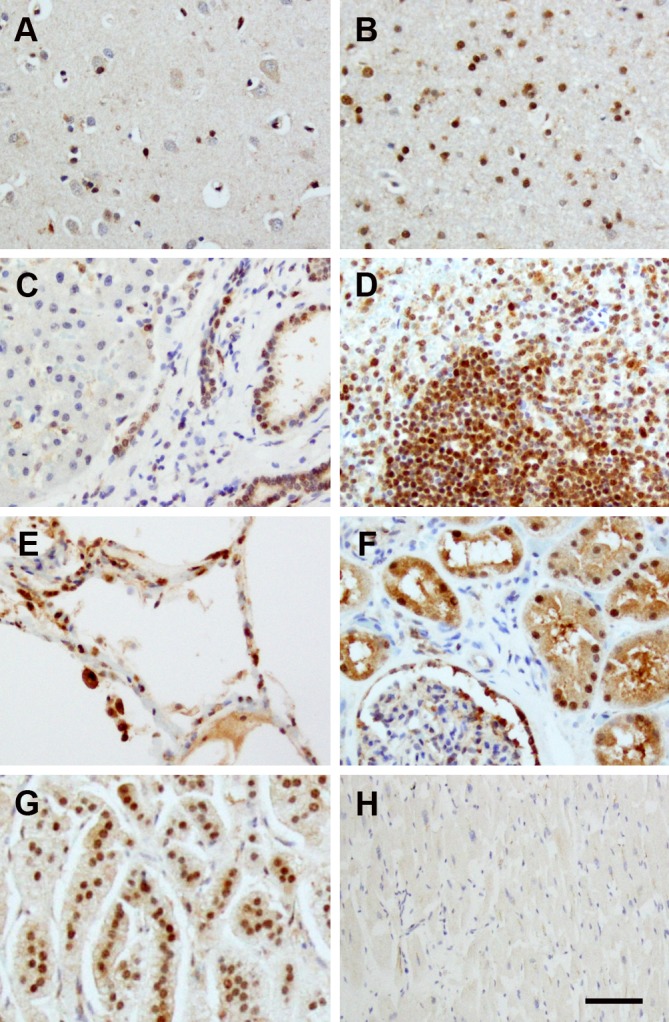
FAM76B expression in normal human organs. (A) brain cortex; (B) brain subcortical white matter; (C) liver; (D) spleen; (E) lung; (F) kidney; (G) adrenal gland; (H) heart. All tissues were stained with MAb No.2. FAM76B was found in most organs, though to different extents. The staining was strongest in the nuclei of lymphocytes in the spleen (D), renal tubular epithelium (F), bile duct (C) and glial cells in the brain (A, B). Intermediate staining was observed in the hepatocytes (C), lung (E), and neurons (A). Glomeruli (F) and cardiac muscle (H) were overall minimally stained. Normal mouse serum was used as a negative control. Primary Abs were detected by a Vectastain ABC Kit with DAB as a substrate. Nuclei were labeled with hematoxylin. Bar = 100 μm.

Lastly, we studied normal human organs for expression of FAM76B, including the brain, lung, liver, kidney, heart, spleen and adrenal gland (Fig 8). FAM76B was found in most organs, though to different extents. In particular, the staining was strongest in the nuclei of lymphocytes in the spleen, renal tubular epithelium, bile duct and glial cells in the brain. Intermediate staining was observed in hepatocytes, alveolar epithelial cells and neurons. Glomeruli and cardiac muscle were overall minimally stained.

## Discussion

In this study, MAbs against hFAM76B were obtained by using hFAM76B recombinant protein expressed in prokaryotic cells and further characterized by ELISAs, Western blot, immunoprecipitation and immunohistochemistry. The epitopes of different MAbs against hFAM76B were further elucidated. Due to the high degree of protein sequence conservation between human and mouse FAM76B, these anti-hFAM76B MAbs also showed high affinity with mFAM76B. In addition, the produced MAbs were used to observe the expression of FAM76B in normal human tissue.

Although six positive stable clones secreting anti-hFAM76B MAbs were identified by direct ELISA, MAb No.4 failed to detect FAM76B protein by Western blotting and immunohistochemistry, possibly due to its lower affinity to FAM76B. An additional lower molecular weight band was detected by Western blot using Mab No. 5 in over expressed FAM76B protein (Fig 2B). This lower band was also present in the endogenous hFAM76b from wild type HEK293, but not in the cell lysate from 293 FAM76B ^-/-^ cells (data not shown). The results suggest that this additional band might correspond to a degradation product of FAM76B protein.

About 20% of proteins produced by the human genome contain single amino acid repeats, among which histidine (His) repeats are uncommonly seen with only 86 proteins bearing stretches of five or more consecutive histidine residues [3, 5, 6]. The functional studies on His-repeats have been very limited so far. Some studies have described His-repeats as nuclear speckle-targeting signals [1,2,3]. In this study, we confirmed the nuclear speckle localization of both human and mouse FAM76B by immunocytochemistry (data not shown for mouse FAM76B). Other studies showed that proteins with histidine stretches had functions related to RNA synthesis [3], and played a major role in development of the nervous system [3, 5]. However, until now, study of poly(His)-containing proteins and their functions has been hampered by a lack of sensitive and specific monoclonal antibodies. The anti-hFAM76B MAbs produced in this study fill this need, and as tools, can facilitate the study the biological functions of FAM76B.

## Supporting Information

S1 Table The sequences of the primers for the amplification of the hFAM76B truncation mutants and the formation of hFAM76B sgRNA. (DOC)
