# Supplementary material for: Production and Characterization of Monoclonal Antibodies against Human Nuclear Protein FAM76B
Source: PLoS One. 2016 Mar 28;11(3):e0152237. doi: 10.1371/journal.pone.0152237 (PMC4809503; doi:10.1371/journal.pone.0152237)
Supplement: S1 Table — (DOC) [file pone.0152237.s001.doc]

**S1 Table**. The sequences of the primers for the amplification of the hFAM76B truncation mutants and the formation of hFAM76B sgRNA

| Primer | Sequence |
| --- | --- |
| F1 of hFAM76B | Forward: 5’- AATCGATATTGTAAAATGTACTTAC-3’ |
|  | Reverse: 5’- ATCTAGATCAAGGAGATGTTAGTATGCT-3’ |
| F2 of hFAM76B | Forward: 5’- AATCGATTATGGACCACCTCAGACCT-3’ |
|  | Reverse: 5’- ATCTAGATCAAGGAGATGTTAGTATGCT-3’ |
| F3 of hFAM76B | Forward: 5’- AATCGATAAAGACCAGCATCATCCA-3’ |
|  | Reverse: 5’- ATCTAGATCAAGGAGATGTTAGTATGCT-3’ |
| F4 of hFAM76B | Forward: 5’- AATCGATTTACAGCAGAGAGACCAGA-3’ |
|  | Reverse: 5’- ATCTAGATCAAGGAGATGTTAGTATGCT-3’ |
| F5 of hFAM76B | Forward: 5’- AATCGATATGGCGGCCTCGGCCCTG-3’ |
|  | Reverse: 5’- ATCTAGATTAATGGCTACTGCTGTGACG-3’ |
| F6 of hFAM76B | Forward: 5’- AATCGATAAAGACCAGCATCATCCA-3’ |
|  | Reverse: 5’- ATCTAGATTACTGCTGTAAGAGACGCTT-3’ |
| hFAM76B-Exon1-sgRNA 1 | Forward:5’-ACCGGTGCAGGCGTACAGGGCCG-3’ |
|  | Reverse:5’- AAACCGGCCCTGTACGCCTGCAC-3’ |
| hFAM76B-Exon1-sgRNA 2 | Forward: 5’- ACCGCACTTGGTGCAGGCGTACA-3’ |
|  | Reverse:5’- AAACTGTACGCCTGCACCAAGTG-3’ |
| hFAM76B-Exon1-sgRNA 3 | Forward: 5’- ACCGACCCAGCGTTATCCTTTCG-3’ |
|  | Reverse:5’- AAACCGAAAGGATAACGCTGGGT-3’ |
| hFAM76B-Exon4-sgRNA 4 | Forward: 5’- ACCGCAATGTGCTTTTGATCGGA-3’ |
|  | Reverse:5’- AAACTCCGATCAAAAGCACATTG-3’ |
